# Parental adjustment to AI learning tools during the preschool-to-primary transition: a qualitative study in China

**DOI:** 10.3389/fpsyg.2026.1758693

**Published:** 2026-03-13

**Authors:** Yiqun Zhang, Wuyuan Guo

**Affiliations:** 1 Department of Education, Graduate School of Sehan University, Sehan University, Yeongam, Republic of Korea; 2 Shenzhen Baoan Haile Experimental School, Shenzhen, China

**Keywords:** AI learning tools, China, parental behavior, preschool–primary transition, qualitative research, theory of planned behavior

## Abstract

This study explores how parents adjust their use of AI learning tools as children move from preschool to primary school. Guided by the Theory of Planned Behavior (TPB), twenty-five semi-structured interviews were analyzed using thematic analysis. Findings reveal that parental attitudes shifted from efficiency-oriented evaluations to context-based judgments of educational fit. Social norms were renegotiated as institutional expectations and peer discussions gained influence. Perceived behavioral control declined due to cognitive overload and resource constraints. Together, these changes led parents to reframe AI tools from routine aids to conditional supports within new schooling structures. The study extends TPB by illustrating how families adapt AI technology use as a form of strategic adaptation within shifting educational and social contexts. By clarifying how parental intentions evolve during institutional transition, it offers practical implications for schools, AI developers, and policymakers seeking to support sustainable and context-sensitive integration of AI in home learning.

## 1 Introduction

The application of artificial intelligence in education (AI) has been widely described as having the potential to enhance learning efficiency and expand educational opportunities. Its values lie in enabling personalized learning and intelligent feedback as well as reshaping instructional practices, assessment routines, and learning support systems (Black and Wiliam, 1998; Zawacki-Richter et al., 2019; Huang et al., 2019). However, empirical studies consistently suggest that although AI learning tools often achieve relatively high rates of initial uptake, long-term use and sustained engagement remain difficult to maintain (Bhattacherjee, 2001; Scherer et al., 2019; Sun et al., 2021). Much of this work has focused on teachers' technology adoption in school settings (Venkatesh et al., 2003), whereas parental decision-making in home-based AI use remains comparatively underexamined.

In this study, AI learning tools refer to adaptive learning applications, AI-powered tutoring systems, and algorithm-based feedback platforms used by children at home to support academic learning. This operationalization is informed by prior classifications of AI applications in education, including intelligent tutoring systems and adaptive learning systems (Holmes et al., 2019; Zawacki-Richter et al., 2019). Within the home learning environment, however, parents are not only resource managers but also key regulators of children's mediated learning (Nikken and Schols, 2015; Neumann, 2017; Livingstone and Blum-Ross, 2020). While prior research has examined parents' mediation of digital learning at home, most studies have focused on screen-time regulation or initial technology acceptance rather than changes in usage patterns over time. In practice, families seldom abandon AI learning tools, but how their use changes during the school-entry stage and what drives these changes remain unclear.

The transition to formal schooling introduces new institutional expectations, homework routines, and performance pressures, which may alter how families evaluate and use AI tools. Understanding these changes is critical for clarifying the sustainability of AI-supported learning within family contexts.

Drawing on the Theory of Planned Behavior (Ajzen, 1991), this study examines parents' discontinuation of AI learning tools during the preschool-to-primary transition. To address this objective, the study poses the following research questions:

RQ1: How do parents' decisions to continue, adjust, or discontinue AI learning tool use evolve during the preschool-to-primary transition?
RQ2: How are these evolving decisions shaped by parents' attitudes, perceived social norms, and perceived behavioral control?

This study is significant in three respects. First, it extends the Theory of Planned Behavior by demonstrating that parental intention is not a one-time decision but an ongoing evaluation shaped by changing school demands and family routines. Second, it offers qualitative evidence from Chinese parents during the preschool-to-primary transition. Third, it provides practical insights for schools, AI developers, and policymakers concerned with sustaining AI-supported learning at home.

## 2 Literature review

### 2.1 Trends and challenges in AI education

The rise of AI has not only driven technological innovation but also accelerated new forms of instructional support and assessment (Holmes et al., 2019). Early expectations centered on the idea that AI could enhance learning efficiency through intelligent feedback, learning analytics, and personalized recommendations (Woolf, 2010). More recently, research in educational technology has moved from traditional computer-assisted instruction toward more adaptive, AI-mediated learning environments (Chen et al., 2020).

Yet increased technological sophistication does not necessarily simplify education itself. Luckin (2018) argues that AI should not be understood as an automated substitute for teachers but rather as a partner in reshaping instructional relationships. Scherer et al. (2019; see reference 5) similarly found that teachers' initial enthusiasm for digital technologies often fails to translate into sustained classroom integration, with long-term engagement declining markedly after the 1st year. Dai et al. (2023) add that the sustainability of AI in education depends on whether learning activities can continue to generate a sense of meaning and efficacy for learners (Hamari et al., 2016). When this motivational linkage weakens, the educational value of the technology diminishes. Consequently, recent research has increasingly focused on the psychological and behavioral mechanisms underlying human–AI interaction, highlighting a shift from technological affordances to human factors. However, while initial adoption has been widely discussed, how AI use is adjusted or sustained over time, particularly within family contexts, remains underexplored.

### 2.2 The role of parents in educational decision-making

Parents play a pivotal role in children's educational decision-making, serving as the primary bridge between home learning and formal schooling (Hoover-Dempsey and Sandler, 1997; Fan and Chen, 2001). Through supervision, communication, and value transmission, they shape children's academic engagement, motivation, and self-regulation (Hill and Tyson, 2009; Spera, 2005). Such decisions, however, are rarely individual or purely rational. From an ecological perspective, families operate within multilayered systems where school expectations, community norms, and cultural beliefs interact to shape educational behavior (Bronfenbrenner, 1979).

In East Asian societies, parental involvement is deeply rooted in a collective understanding of education as both a moral duty and a route to intergenerational advancement (Bray and Kwo, 2014). This cultural orientation positions parents not as passive supporters but as active planners who continuously evaluate the potential risks, legitimacy, and long-term returns of educational choices (Hu and Yeung, 2019). Family-based educational investment thus functions as a strategy for maintaining social mobility and fulfilling familial responsibility. Within this context, decision-making about learning resources—whether tutoring, digital platforms, or AI-based programs—reflects a culturally embedded desire to optimize the child's developmental trajectory rather than a simple pursuit of academic performance (Bronfenbrenner, 1979; Grenfell, 2009).

### 2.3 Parents and AI education

Within the home learning environment, AI learning tools have become a significant medium through which parents manage and interpret their children's learning experiences (Livingstone and Blum-Ross, 2020; Chaudron et al., 2018). As key decision-makers, parents determine not only whether such tools are adopted but also how they are maintained and adjusted over time. Their engagement thus shapes the sustainability and educational meaning of AI use in everyday family routines (Lin et al., 2023). Studies further indicate that parental trust, perceived usefulness, and perceived risks jointly inform their attitudes toward AI learning tools and their patterns of use (Ata-Aktürk and Akman, 2024).

However, existing research has primarily examined initial adoption and mediation practices, leaving relatively little understanding of how and why parents subsequently reduce or recalibrate AI use over time. In managing the tensions between control and dependence that AI use can create, parents often employ strategies of parental mediation. Such strategies combine restriction with guided use and reflect a cautious stance toward technological exposure while simultaneously negotiating the child's autonomy in learning (Engel et al., 2019). As conceptions of digital parenting evolve, parents are increasingly positioned not as passive monitors but as collaborative partners, and AI-based learning becomes a co-constructed activity between parent and child (Venkatesh et al., 2012; Jeffery, 2025).

Despite growing recognition of the importance of home involvement in technology adoption, the mechanisms through which parents reduce the frequency of AI use remain underexplored. Understanding parental adjustment in AI-mediated learning therefore requires moving beyond a simple adoption lens toward a dynamic account of how beliefs, social expectations, and perceptions of control interact over time.

### 2.4 AI education in the Chinese policy context

Since the launch of the Education Informatization 2.0 Action Plan in 2018, China's drive toward educational digitalization has become increasingly institutionalized and systematized (Yan and Yang, 2020). Artificial intelligence has been incorporated into the national strategy for education modernization and is framed as a mechanism for promoting both educational equity and instructional quality (Aydin, 2024).

However, as Selwyn (2021) argues, the diffusion of educational technology is fundamentally a process of establishing legitimacy. For a technology to persist in practice, it must gain recognition at both discursive and institutional levels. Schools play a pivotal mediating role in this process: resource provision, teacher training, and instructional framing shape parental trust in AI and influence how families interpret its appropriate use (Hu and Yeung, 2019; Wang and Zhao, 2021). Policy discourse is gradually interpreted at the family level, shaping everyday decisions about homework, supplementary learning, and the perceived appropriateness of AI use.

At the same time, Chinese parents' educational decisions are shaped by the dual pressures of institutional expectations and the culturally embedded logic of educational competition (Hu and Yeung, 2019). Consequently, the use of AI learning tools is best understood as a behavioral negotiation in which parents weigh perceived benefits, respond to social expectations, and assess their capacity to manage technology use within family routines. Understanding this process requires a framework that captures how attitudes, norms, and control jointly guide parental adjustment during the preschool-to-primary transition.

### 2.5 Theoretical framework: the theory of planned behavior

To interpret the process of parental negotiation, the present study adopts the Theory of Planned Behavior (TPB). The TPB posits that behavioral intention is jointly predicted by attitudes, subjective norms, and perceived behavioral control (Fishbein and Ajzen, 2010). Compared with traditional technology acceptance models, TPB offers explanatory reach not only for initial adoption but also for sustained use, adjustment, and even discontinuation (Conner and Armitage, 1998; Ajzen, 2002).

**Figure 1:**
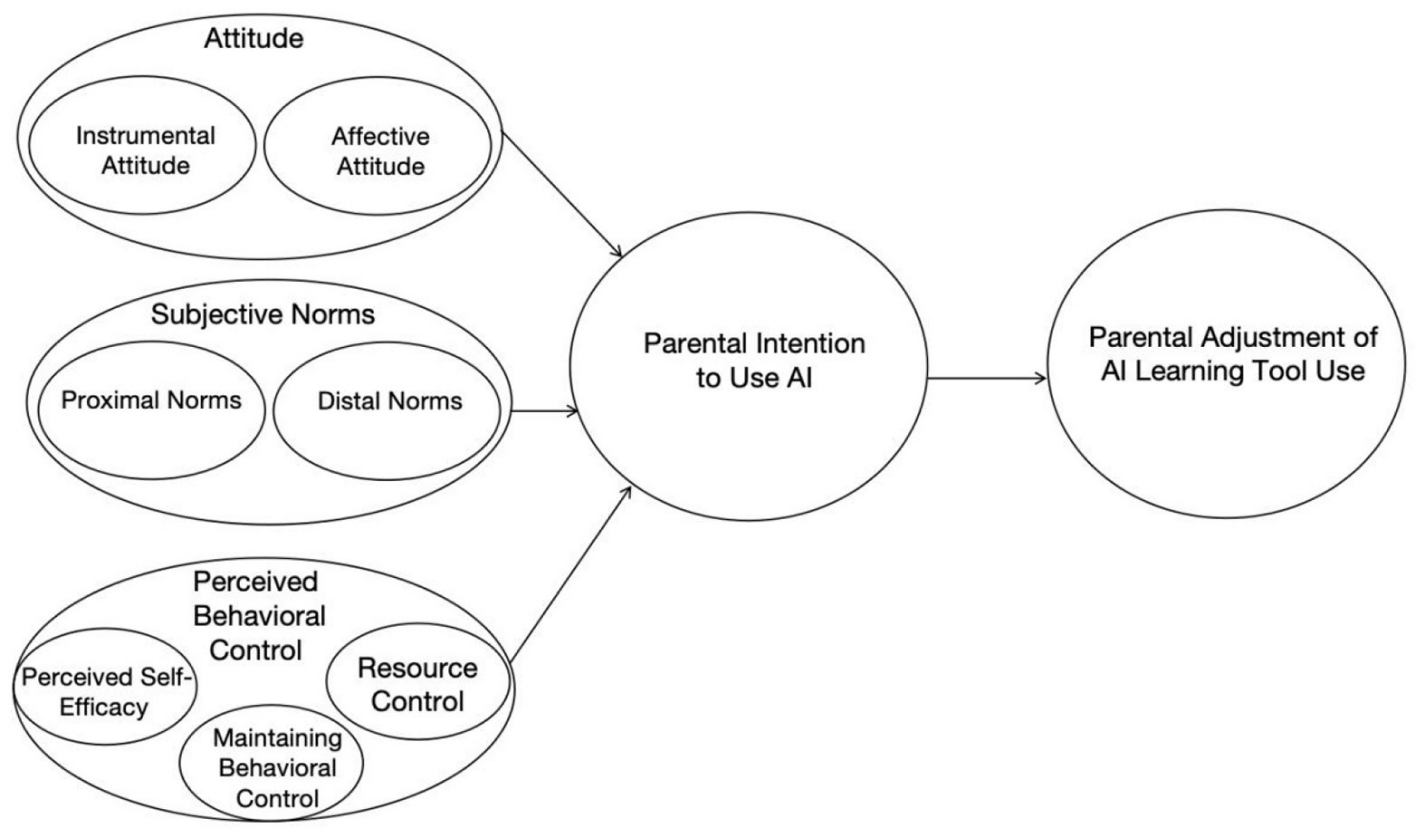
Contextualized TPB framework of parental adjustment to AI learning tools.

This framework is particularly suited to the present study because parents' engagement with AI learning tools involves ongoing evaluation, adaptation, and sometimes reduction of use rather than one-time adoption. In the present study, attitudes were operationalized as parents' instrumental evaluations of the effectiveness and contextual fit of AI learning tools, as well as their affective experiences during everyday use. Subjective norms referred to perceived expectations from teachers, other parents, and broader institutional and media discourses that shaped judgments about appropriate educational practice. Perceived behavioral control encompassed parents' assessments of time availability, cognitive demands, supervision burden, and emotional costs embedded in daily family routines. These three components jointly shape parents' willingness to adopt, sustain, or discontinue AI learning tools. Guided by this framework, TPB informed both the design of the interview protocol and the coding structure for subsequent analysis. The conceptual framework guiding this study is presented in Figure 1.

## 3 Materials and methods

### 3.1 Participants and Sampling

This study focused on parents of first-grade primary school children and examined how they adjusted their use of AI learning tools as their children transitioned from preschool into formal schooling. All participants were mothers who had experience using AI learning tools during the preschool-to-primary transition.

Participants were recruited through purposive sampling from a Chinese public primary school in Changchun, Jilin Province. The invitation was distributed via the class teachers through the official parent communication channel; parents contacted the research team directly if interested. The inclusion criteria required that participants (a) were the primary caregiver of a first-grade child and (b) had prior experience using AI learning tools during the preschool-to-primary transition. Interested parents contacted the research team voluntarily. A total of 30 parents expressed interest in participating, and 25 mothers ultimately completed the interviews. Five parents declined due to scheduling constraints.

Approximately two-thirds of the mothers held a bachelor's degree or higher, while one-third had below-bachelor education levels. The AI learning tools most frequently used by participants included early literacy apps, English learning apps, and math learning apps. Most participants reported frequent use of these tools during the preschool stage, typically 4 to 5 times per week, which declined to 1 or 2 times per week after school entry. Data collection continued alongside preliminary analysis. After the 22nd interview, no substantively new codes emerged during iterative coding. Three additional interviews were conducted to confirm thematic redundancy, after which data collection was concluded, indicating theoretical saturation. Although fathers were also eligible, those who volunteered and completed interviews were all mothers. Demographic characteristics of the participants are summarized in Table 1.

**Table 1:** Demographic information of participants.

**ID**	**Gender**	**Education level**	**Type of AI learning tool**	**Frequency (preschool stage)**	**Frequency (primary stage)**
P01	Female	Below bachelor	Literacy	4–5 times/week	1–2 times/week
P02	Female	Bachelor	English	Daily	1–2 times/week
P03	Female	Bachelor	Reading	4–5 times/week	Once/week
P04	Female	Master	Integrated learning	4–5 times/week	Occasionally
P05	Female	Below bachelor	Literacy	4–5 times/week	Once/week
P06	Female	Bachelor	English	4–5 times/week	1–2 times/week
P07	Female	Bachelor	Reading	4–5 times/week	Once/week
P08	Female	Below bachelor	Integrated learning	3–4 times/week	None
P09	Female	Bachelor	Literacy	4–5 times/week	Once/week
P10	Female	Bachelor	English	4–5 times/week	1–2 times/week
P11	Female	Master	Reading	Daily	Once/week
P12	Female	Below bachelor	Integrated learning	4–5 times/week	Once/week
P13	Female	Bachelor	English	4–5 times/week	Once/week
P14	Female	Bachelor	Literacy	4–5 times/week	1–2 times/week
P15	Female	Master	Reading	5 times/week	Once/week
P16	Female	Below bachelor	Integrated learning	3–4 times/week	None
P17	Female	Bachelor	English	4–5 times/week	1–2 times/week
P18	Female	Bachelor	Reading	4–5 times/week	Once/week
P19	Female	Bachelor	Literacy	4–5 times/week	1–2 times/week
P20	Female	Below bachelor	Integrated learning	3–4 times/week	None
P21	Female	Bachelor	English	4–5 times/week	Once/week
P22	Female	Master	Reading	Daily	Once/week
P23	Female	Bachelor	Literacy	4–5 times/week	1–2 times/week
P24	Female	Bachelor	Integrated learning	4–5 times/week	Occasionally
P25	Female	Below bachelor	English	3–4 times/week	None

*P* = Participant; “Preschool Stage” and “Primary Stage” refer to children's learning periods before and after entering Grade 1.

### 3.2 Data collection

Informed consent was obtained from all participants prior to the interviews. A total of 25 semi-structured interviews were conducted by telephone between June and August 2025. Each interview lasted approximately 40 to 60 min. The interviews were conducted by the first author, who had no prior personal relationship with the participants before recruitment.

The interview protocol was organized around the dimensions of the Theory of Planned Behavior (Ajzen, 1991), including attitudes (instrumental and affective), subjective norms (influences from family members, peers, and teachers), and perceived behavioral control (prior experience, resource constraints, and time demands). The interview guide was developed based on TPB literature and prior studies on educational technology use, and was refined through iterative discussion within the research team.

Sample interview questions included:

“How did your use of AI educational tools change after your child entered primary school?”, “What factors influenced your decision to continue, reduce, or discontinue their use?”, and “What challenges did you encounter when supporting your child's use of these tools?”

All interviews were audio-recorded with permission and transcribed in full into Chinese immediately following the session. All personal identifiers were removed at the point of transcription and analysis to protect confidentiality. The semi-structured format allowed for theoretical consistency while also capturing variation in parental experiences across different household and schooling contexts. The study was conducted in accordance with institutional ethical guidelines. The full interview guide is available in Supplementary Data Sheet 1.

### 3.3 Data analysis

Prior to analysis, the research team conducted a line-by-line accuracy check of all transcripts to ensure fidelity. Quotations from parents that appear in this manuscript were checked bilingually. Only the cited excerpts were translated into English, and all coding and interpretation were conducted in Chinese.

Deductive thematic analysis approach was employed by using the three core dimensions of the Theory of Planned Behavior (TPB) as the initial coding frame. At the same time, sub-themes were refined inductively to capture context-specific patterns emerging from participants' narratives. Two members of the research team independently generated preliminary codes, followed by iterative comparison and discussion to reach thematic consensus and enhance reliability. Coding was managed manually using structured spreadsheets to organize themes and sub-themes. This process yielded a set of main themes and sub-themes that describe parental reasoning and adjustment as they reduced the frequency of AI learning tool use.

To ensure trustworthiness, we followed Lincoln and Guba's (1985) criteria for qualitative rigor. Peer debriefing sessions were conducted within the research team to critically examine emerging interpretations. Member checking was carried out by sharing brief summaries of the preliminary themes with a subset of participants to confirm whether the interpretations accurately reflected their experiences. Minor clarifications were incorporated based on their feedback. Member checking involved five participants, who reviewed a one-page summary of themes and confirmed overall fit; their feedback led to minor wording clarifications without changing the thematic structure. Intercoder reliability was further ensured through repeated coding comparison and consensus validation between the two researchers.

The research team consists of scholars in educational psychology with research experience in AI-supported learning. Reflexive discussions were conducted throughout the analytic process to consider potential assumptions about AI educational tools and parental decision-making, thereby minimizing interpretive bias.

## 4 Findings

The findings are organized around the three core dimensions of the Theory of Planned Behavior (TPB): attitudes, subjective norms, and perceived behavioral control. Table 2 provides an overview of the key themes and representative quotations. In addition to these dimensions, an emergent pattern of conditional intention is presented.

**Table 2:** Summary of themes and representative quotations.

**TPB dimension**	**Subtheme**	**Representative Quote**
Attitudes	Instrumental attitude: from school readiness support to comparative reassessment	“Online learning just doesn't have that feeling. In a real classroom she'll even try to keep up with her classmates.” (P14)
	Affective attitude: from co-learning warmth to emotional fatigue	“If I wasn't next to her, she wouldn't start. I'd have to keep reminding her, and then we'd argue.” (P13)
Subjective norms	Proximal norms: from shared commitment to role ambivalence	“The school already has a system. If I keep teaching at home she'll get confused about who to listen to.” (P11)
	Distal norms: institutional authority and the reconfiguration of educational expectations	“In primary school the teacher doesn't really bring up these apps.” (P15)
Perceived behavioral control	Perceived self-efficacy: from manageable simplicity to cognitive overload	“It used to help me, but now it just takes more time to figure out how to use it right.” (P22)
	Maintaining behavioral control: control erodes under layered demands	“If I tell her to study, she gets upset.” (P08)
	Resource control: reassessing cost–benefit and lowering frequency	“There are more in-person classes now, and they're not even that much more expensive.” (P19)
Emergent pattern	Conditional intention and future adaptation	“If the app could follow the school textbook and not need me sitting next to her, I would use it again.” (P09)

### 4.1 Attitudes

#### 4.1.1 Instrumental attitudes: from school readiness support to comparative reassessment

During the preschool stage, most parents (*n* = 19) commonly described AI learning tools as a practical means of preparing children for the rhythms and expectations of primary school. Structured lessons and instant feedback made learning progress visible in the home and enabled children to acquire basic literacy and numeracy skills. Many parents reported that these tools eased their anxieties about school entry and enhanced confidence in their own educational efforts.

“One year before primary school I had her do about 20 min of AI classes every day. I felt it helped her adjust faster once formal classes started. It was convenient, she picked things up quickly, and I didn't have to search everywhere for materials”. (P06)

After school entry, a majority of participants (*n* = 15) reported that their evaluations of AI learning tools became more cautious and comparative. They increasingly mentioned the advantages of in-person classroom settings, particularly teacher oversight, peer interaction, and the collective atmosphere that helps sustain attention and discipline.

“When the teacher is right there, she listens carefully. Online learning just doesn't have that feeling. In a real classroom she'll even try to keep up with her classmates.” (P14)

Parents also reported that AI learning tools were less effective in subjects requiring embodied demonstration or real-time correction. Several mothers (P03, P22, P25) emphasized that skills such as calligraphy, drawing, and oral reading were considered to depend on immediate feedback and detailed guidance on posture, hand position, pacing, and expression—forms of instruction they felt AI platforms could not fully provide.

“For calligraphy, AI can only tell her to trace. Nobody corrects how hard she presses or how she's holding the brush.” (P22)

After school entry, parents reduced AI use to specific contexts. This reflected a reassessment of instrumental utility, marking a shift toward selective and efficiency-driven use. Taken together, these accounts illustrate how instrumental attitudes were recalibrated in response to changing academic demands, rather than simply reversed.

#### 4.1.2 Affective attitudes: from co-learning warmth to emotional fatigue

In the preschool stage, many parents (*n* = 17) often described AI learning tools as a shared activity that encouraged parent–child interaction. Evening or weekend sessions were viewed as relaxed and cooperative occasions during which parents and children watched lessons together and completed small tasks as part of family time.

“At the beginning he was always excited. We watched the AI lessons together, and he would answer loudly like it was his own little class.” (P07)

Positive engagement from the child reinforced parental motivation and created a sense of accomplishment and connection. Over time, however, this atmosphere of co-learning diminished as children entered primary school. More than half of the mothers (n=14) noted that although they expected greater independence, their children rarely used AI tools on their own. Parents described needing to remind, supervise, or persuade the child repeatedly.

“After first grade I wanted her to do it by herself. But if I wasn't next to her, she wouldn't start. I'd have to keep reminding her, and then we'd argue. At some point I just thought, let the teacher handle it.” (P13)

Parents observed that this repeated prompting often led to frustration and conflicts. Several mentioned reducing or suspending AI sessions to avoid arguments or emotional exhaustion. Some families shifted toward school-based or in-person instruction, explaining that it reduced daily tension and required less direct supervision.

“It just became tiring. I preferred her to study at school. The teacher keeps an eye on her, and we don't have to fight about it anymore.” (P20)

Overall, parents described a gradual change in emotional experience. AI learning increasingly became linked with stress and fatigue. These affective shifts were closely intertwined with instrumental reassessments, as diminished emotional rewards reinforced parents' decisions to scale back AI use. These tensions pointed to recurring struggles over children's autonomy and parental regulation, which later informed our interpretation in the Discussion.

### 4.2 Subjective norms

#### 4.2.1 Proximal norms: from shared commitment to role ambivalence

During the preschool stage, many families (*n* = 22) shared the belief that early preparation would help children transition smoothly to Grade 1. The use of AI learning tools was thus regarded as part of responsible parenting. Through informal comparison among parents, this practice gradually acquired a moral dimension associated with diligence and foresight.

“Back then I felt there was nothing wrong with starting early—learning some pinyin, recognizing characters, making Grade 1 easier. It felt worthwhile.” (P05)

After school entry, parents‘ understanding of educational responsibility shifted. Confidence in teachers' professional authority and the structure of the school curriculum led many to scale back home-based use of AI learning tools and prioritize formal instruction.

“Now that she's in school I think the teacher should be in charge. The school already has a system, If I keep teaching at home she'll get confused about who to listen to.” (P11)

However, this trust did not fully dispel anxiety. Peer comparison among parents persisted even as they deferred to the school. Several mothers (P01, P18, P21) described a tension between trusting the teacher's professional judgment and worrying that their child might fall behind peers whose families continued supplementary learning at home.

“Part of me thinks the teacher will handle it, but then I hear other parents saying they‘ve enrolled in extra programs. I start to worry she'll fall behind. I don't want to keep teaching at home. It's tiring, but I also don't dare to stop using AI entirely.” (P18)

Parents thus reported a state of role ambivalence. While acknowledging the professional division of labor between home and school, they remained responsive to the implicit pressure of social comparison within parent networks. As a result, most continued limited engagement with AI learning tools even as overall frequency declined.

#### 4.2.2 Distal norms: institutional authority and the reconfiguration of educational expectations

During the preschool stage, teachers often encouraged parents to extend learning at home. Many parents (*n* = 14) recalled being advised to continue picture books, practice activities, or AI learning tools, which were widely regarded as appropriate forms of early preparation for school entry.

“The kindergarten teacher would say, ‘Read more together at home, it'll help in Grade 1.' So I thought using AI learning tools at home was part of helping her.” (P04)

After children entered primary school, educational authority gradually shifted from the household to the formal school system. Teachers no longer recommended external tools and instead asked parents to “follow the school curriculum”. Parents (n = 18) described this as a signal to reduce the use of AI-based learning resources.

“In primary school the teacher doesn't really bring up these apps. Since the teacher doesn't mention them, I don't ask. I just quietly let him use some software at home, thinking it's better than nothing.” (P15)

The national policy aimed at reducing homework load and curbing off-campus tutoring further reinforced this restraint. Teachers were careful not to endorse after-school learning, both to comply with regulations and to avoid appearing to encourage academic intensification. Parents reported becoming more cautious about home-based instruction and preferred not to cross perceived institutional boundaries.

At the same time, commercial platforms and online media sent competing signals. Through short videos and targeted promotions, parents were exposed to narratives emphasizing “AI empowerment,” “early advantage,” and “winning at the starting line,” which reproduced a sense of ongoing competition (Livingstone and Blum-Ross, 2020).

“On TikTok everyone says kids who use AI learn faster. It makes you feel like you're already behind. But when you try it, it's not that magical. It's not useless, but definitely does notwork out like the videos say.” (P21)

Parents thus navigated two conflicting normative directions. Institutional norms encouraged alignment with school expectations, while market-oriented discourse promoted continued acceleration. Many families described maintaining AI learning tools as a supplementary activity—still present, but deliberately limited in scope and frequency.

### 4.3 Perceived behavioral control

#### 4.3.1 Perceived self-efficacy: from manageable simplicity to cognitive overload

During the preschool stage, most parents (*n* = 20) generally experienced the use of AI learning tools as straightforward and easy to manage. The interfaces were simple, animated content captured children's attention, and built-in feedback allowed parents to monitor progress with little effort. AI learning was thus perceived as a controllable and efficient supplement to home instruction.

“It wasn't complicated. It just felt easy to use.” (P09)

After children entered primary school, this sense of control weakened. The curriculum became more demanding, and differences among textbook versions required parents to compare multiple platforms, verify alignment with classroom lessons, and make frequent adjustments. The rapid expansion of AI tools and continuous content updates also created a sense of informational overload.

“There are too many versions. I keep trying different ones because I'm worried the AI teaches things differently from school. Sometimes I spend more time checking than she does learning.” (P14)

Parents described an increasing need to evaluate and intervene, which made AI learning feel less manageable than before. What had once been framed as a low-effort aid gradually turned into an activity requiring constant supervision and decision-making. Several parents reported (*n* = 7) that the growing cognitive burden diminished their confidence in sustaining regular use.

“It used to help me, but now it just takes more time to figure out how to use it right.” (P22)

Overall, parents' experiences reflected a gradual shift from perceiving AI learning tools as effortless supports to viewing them as cognitively demanding tasks that required continual monitoring. These accounts suggest a decline in perceived self-efficacy, as parents felt less confident in their ability to manage AI use effectively within increasingly complex academic environments.

#### 4.3.2 Maintaining behavioral control: control erodes under layered demands

As children entered primary school, many parents (*n* = 19) reported feeling “unable to keep up” with the demands of maintaining consistent AI use. Academic requirements multiplied, schedules became tighter, and household routines increasingly overlapped with school expectations, making it difficult to sustain a stable AI learning schedule.

“There's just too much now—reading, picture-based composition, English, PE, calligraphy. I can't keep up with all of it.” (P14)

Time pressure was only one aspect. As children's autonomy and peer awareness increased, parental reminders frequently led to resistance and conflict.

“She says her classmates are using a different app and doesn't want to keep using this one. If I tell her to study, she gets upset. Either I remind her every day and she ignores me, or we argue. I can't force it.” (P08)

Parents described continuing to recognize the value of AI learning tools but found it increasingly difficult to maintain regular use. AI learning shifted from a fixed daily activity to an occasional option used only when time, energy, or mood allowed.

Overall, parents revealed that behavioral control over AI learning gradually weakened under the combined pressures of school workload, time constraints, and family interaction dynamics.

#### 4.3.3 Resource control: reassessing cost–benefit and lowering frequency

During the preschool stage, parents (*n* = 10) widely viewed AI learning tools as cost-effective. In-person classes for young children were expensive and difficult to access, while AI platforms offered affordable subscriptions and extensive content.

“Back then AI courses were really cheap. A month's fee covered so much. An in-person class would cost hundreds for one session.” (P08)

By the early primary years, this perception had shifted. Although subscription fees remained low, parents reported that effective use required constant supervision, repeated prompting, and emotional regulation(P07, P14, P25). The “cost” of AI use came to include time, energy, and relational strain.

“It looks cheap, but it drains you. I have to sit next to him every day or he won't do it. The effort is huge.” (P14)

At the same time, small-group in-person classes became more available and were described as more manageable alternatives, offering direct feedback and external discipline.

“There are more in-person classes now, and they're not even that much more expensive. The teacher watches her, which is better than me chasing her, and I don't have to worry.” (P19)

Many families thus re-evaluated their investment priorities. The perception of AI as a “high-value, low-cost” option was replaced by experiences of “high effort, uncertain return,” while offline instruction became a more practical and predictable choice. Parents reported redirecting their limited time and emotional energy toward settings that felt more sustainable and externally managed.

### 4.4 Others: conditional intention and future adaptation

Beyond the three core dimensions of the TPB, a majority of parents (*n* = 14) displayed a future-oriented pattern of decision-making that extended beyond immediate behavioral control. Many families did not regard the reduction of AI use as a complete withdrawal but as a temporary adjustment. They described a conditional willingness to resume use if contextual barriers such as time pressure, limited school alignment, or the need for constant supervision were resolved.

Several parents explicitly expressed this conditional openness:

“If the app could follow the school textbook and not need me sitting next to her, I would use it again.” (P09)

“It's not that I dislike AI—it just doesn't fit now. Maybe when she's older and more self-disciplined, it will help again.” (P22)

This sense of postponement prevented the psychological closure associated with abandonment and transformed discontinuation into a temporary withdrawal rather than a final exit. Parents also differentiated between the present misfit and the future promise of AI. They emphasized that technological improvement, better integration with school curricula, or reduced parental involvement could restore their willingness to re-adopt:

“If schools actually used similar systems, I think it would make more sense for us to continue at home.” (P18)

This conditional stance indicates that discontinuation was shaped less by stable rejection and more by expectations of future normative support and improved behavioral control. This finding suggests that intention among parents is not a fixed or one-time decision but a dynamic and context-sensitive process that is continuously revised and reactivated as educational and family contexts evolve.

## 5 Discussion

Guided by the Theory of Planned Behavior (TPB), this study examined how parents adjusted their use of AI learning tools during the transition from preschool to primary school. The findings indicate that reduced frequency of use should not be interpreted as declining motivation or technological failure. Rather, it reflects an active strategy by which parents reconfigure learning arrangements in response to shifting educational contexts, social expectations, and resource demands (Ajzen, 1991; Bandura, 2001).

Parental attitudes evolved from efficiency-oriented evaluations to contextually grounded judgments of fit. During the preschool period, AI learning tools were seen as low-risk and high-yield supports that offered immediate feedback and visible progress (Hamari et al., 2016; Shute, 2008). Once formal schooling began, however, parents' evaluations became closely tied to institutional legitimacy and social appropriateness (Li et al., 2025; Zhang et al., 2025). The inability of AI tools to provide real-time correction in skill-based domains such as calligraphy, drawing, or oral expression revealed the limits of technology–context congruence (Luckin, 2018). This extends TPB by demonstrating that parental evaluations of AI are tied to specific learning contexts, such as skill-based vs. content-based tasks, rather than reflecting a general approval or disapproval of the technology.

Parental norms were reconstructed through negotiation between two competing systems of expectation. During the preschool stage, using AI tools symbolized responsible parenting and diligence (Bandura, 2001; Hsu and Chen, 2023). With the onset of primary education, the authority of schools and teachers redefined what counted as legitimate learning. Institutional signals, reinforced by policy, encouraged compliance, while online platforms continued to promote acceleration narratives such as “AI empowerment” and “winning at the starting line” (Yu, 2024; Guo et al., 2025). Parents therefore balanced symbolic participation in supplemental learning with performative compliance with school norms, illustrating that subjective norms are dynamic, socially constructed, and contextually embedded (Valkenburg et al., 2022; Üztemur et al., 2025). In this context, subjective norms emerged from tensions between institutional expectations and platform-mediated acceleration discourses in China. This finding suggests that subjective norms in educational technology contexts operate across institutional and market levels, extending TPB beyond single-source normative influence.

Shifts in perceived behavioral control explained how intentions translated into practice. Initially, AI learning tools were manageable and effective. Yet as schooling demands increased, parents faced greater cognitive load and decision fatigue, alongside emotional negotiation with more autonomous children. AI use became a cognitively and emotionally demanding task rather than a low-effort aid (Ryan and Deci, 2024). This demonstrates that behavioral control in digital learning is constrained not only by time or technology but also by the cognitive and emotional costs embedded in family routines (Fan and Hui, 2025). This extends TPB by highlighting how perceived control in family settings is shaped by relational and emotional labor, not only resource availability.

Finally, perceptions of cost evolved from financial to multidimensional assessments involving effort, emotion, and sustainability. Parents redirected limited time and energy toward environments that minimized strain and maximized clear outcomes (Obeid et al., 2024). The decline in AI use thus represents a repositioning of technology within the family learning ecology rather than a withdrawal. By integrating contextual fit, normative negotiation, and resource governance, this research deepens understanding of how behavioral intentions are sustained, adapted, or constrained within institutionalized educational environments. Rather than treating intention as a one-time adoption decision, this study illustrates how TPB processes unfold dynamically within family-level and relational contexts.

## 6 Theoretical and practical implications

This study employs Theory of Planned Behavior (TPB) to explain how parents adjust their children's AI learning tools as educational contexts shift. The preschool-to-primary transition triggered a process of strategic repositioning, during which parents re-evaluated the instrumental and emotional meanings of AI use. The findings show that parental intention is not a single, fixed judgment but an evolving process shaped by contextual fit, institutional cues, and resource constraints. Rather than treating intention as a one-time adoption decision, this study demonstrates that TPB operates within a family-level ecology in which multi-level norms (institutional, peer, and market) simultaneously shape parental reasoning. In addition, perceived behavioral control in this context extends beyond technical competence to include cognitive load and emotional regulation embedded in daily family routines. These insights indicate that TPB enables the understanding of how parents' intentions change as educational environments evolve, thereby extending its application from individual technology adoption to relational and context-sensitive family decision-making.

Practically, the findings highlight the need to design family-centered AI learning environments that align with school expectations and reduce parental cognitive and emotional burdens. For schools, clearer communication regarding the role of home-based AI tools may reduce parental ambivalence during the transition period. For AI developers, closer alignment with school curricula and reduced supervision demands may enhance sustained engagement. Policymakers and all stake holders should integrate teacher feedback, provide clearer guidance, and promote flexible, age-appropriate use to support sustainable engagement in home learning. Importantly, policy initiatives should also consider how regulatory signals and public discourse shape parental perceptions of legitimacy and appropriateness in everyday practice.

## 7 Limitations and future research directions

This study is limited by its single-city sample and reliance on self-reported qualitative data. All interviews were conducted by telephone rather than face-to-face, which may have restricted access to non-verbal cues and contextual information that could enrich interpretation. In addition, parents were asked to reflect retrospectively on their use of AI tools during the preschool period, which may introduce recall bias. Moreover, all participants were mothers. While this reflects the caregiving structure within the sampled context, it may limit understanding of how AI-related decisions are negotiated within the broader family system.

Future research should include diverse socioeconomic contexts and adopt longitudinal or mixed-method designs to trace how parental adjustment evolves over time. Including multiple caregivers within the same household may further illuminate intra-family negotiation processes in digital learning decisions. Cross-cultural comparisons could further reveal how cultural norms and policy environments shape digital parenting practices. Examining the interaction between parental regulation, children's learning outcomes, and the design of AI systems will provide deeper insight into sustainable technology use in family education.

## 8 Conclusion

The study found that the decline in AI use does not signal rejection but a strategic recalibration balancing institutional legitimacy, family relations, and manageability. Parental decision-making reflects a negotiated process shaped by attitudes, norms, and perceived control across changing contexts.

Understanding these dynamics enriches TPB's explanatory power and offers practical insights for designing age-appropriate, emotionally sustainable AI learning practices that support both educational quality and family educational wellbeing. If these contextual, normative, and resource-related dynamics are overlooked, initiatives promoting AI learning tools may unintentionally increase parental burden, intensify normative tensions, and undermine sustained engagement in home learning.

## Data Availability

The datasets presented in this article are not readily available because the dataset contains sensitive qualitative interview data involving human participants. According to the informed consent and institutional guidelines, the raw data cannot be shared publicly or upon request. Only aggregated, anonymized findings are available in the article.
