# Supplementary material for: Parental adjustment to AI learning tools during the preschool-to-primary transition: a qualitative study in China
Source: Front Psychol. 2026 Mar 13;17:1758693. doi: 10.3389/fpsyg.2026.1758693 (PMC13021866; doi:10.3389/fpsyg.2026.1758693)
Supplement: Supplementary file 1 [file Data_Sheet_1.pdf]

Interview Protocol for Semi-Structured Parent Interviews

Section 1. Background Information

1. What is your age range?
2. What is your educational background?
3. What is your current occupation?
4. At which educational stage is your child currently enrolled? (Middle kindergarten / Upper kindergarten / Grade 1)

Section 2. Child’s Learning Activities and Exposure to Educational Technologies

5. Through which types of learning activities does your child typically engage? (e.g., kindergarten curriculum, after-school classes, online learning)
6. Has your child used any AI-based educational products? If so, please specify the names of those you know or have used.
7. How did you first learn about these products? Who recommended them, or through which channel did you become aware of them?

Section 3. Attitudes Toward AI Educational Tools

8. In your view, what advantages or positive features do these AI educational tools offer? (e.g., engagement, personalization)
9. Are there any aspects of these tools that you find unsatisfactory or inappropriate for your child?
10. How does your child respond when using these tools? Do they enjoy them or request to use them independently?
11. How do you personally feel about these tools? Have they had any emotional or practical influence on your family life?

Section 4. Subjective Norms

12. How do other family members (such as your child, spouse, or grandparents) perceive these tools?
13. Do your peers, colleagues, or other parents use similar tools? If so, does their experience or evaluation influence your decisions?
14. Have teachers recommended such tools or assigned related tasks?

Section 5. Perceived Behavioral Control

15. To what extent do you feel capable of assisting your child in using these tools effectively?
16. Have you encountered any challenges (e.g., time constraints, technical problems, operational difficulties)?
17. Do you have concerns that AI tools might reduce parent-child interaction or substitute interpersonal communication?
18. When using AI tools, does your child require parental supervision or guidance? Has this influenced your usage decisions?
19. What do you find most challenging when accompanying your child during AI-tool use? Has this contributed to any interruptions or discontinuation?

Section 6. Decision-Making Processes and Changes Over Time

20. Did you make a one-time decision to use or not use these tools, or did you evaluate them after trying?

21. Were there specific events that changed your intention to use or continue using them? (e.g., changes in your child’ s reactions, conflicts with school assignments)
22. Compared with the initial period of use, how do you currently view these tools?
23. Have you discontinued the use of any AI educational tool at any point? What were the reasons?
24. What made you feel the tool was “no longer suitable” ? Were there specific events or contextual changes? (e.g., transition to primary school, tighter schedules, lower-than-expected effectiveness)
25. Although you are not currently using these tools, would you consider resuming use if they become more aligned with the curriculum, easier to operate, or require less parental involvement?

Section 7. Additional Considerations

26. Are there any other factors — beyond those discussed above — that you believe influenced your decision to continue or discontinue the use of AI educational tools?
